# Structure of an Enzyme-Derived Phosphoprotein Recognition Domain

**DOI:** 10.1371/journal.pone.0036014

**Published:** 2012-04-24

**Authors:** Christopher A. Johnston, Chris Q. Doe, Kenneth E. Prehoda

**Affiliations:** 1 Institute of Molecular Biology, University of Oregon, Eugene, Oregon, United States of America; 2 Institute of Neuroscience and Howard Hughes Medical Institute, University of Oregon, Eugene, Oregon, United States of America; Virginia Commonwealth University, United States of America

## Abstract

Membrane Associated Guanylate Kinases (MAGUKs) contain a protein interaction domain (GK^dom^) derived from the enzyme Guanylate Kinase (GK^enz^). Here we show that GK^dom^ from the MAGUK Discs large (Dlg) is a phosphoprotein recognition domain, specifically recognizing the phosphorylated form of the mitotic spindle orientation protein Partner of Inscuteable (Pins). We determined the structure of the Dlg-Pins complex to understand the dramatic transition from nucleotide kinase to phosphoprotein recognition domain. The structure reveals that the region of the GK^dom^ that once served as the GMP binding domain (GBD) has been co-opted for protein interaction. Pins makes significantly more contact with the GBD than does GMP, but primarily with residues that are conserved between enzyme and domain revealing the versatility of the GBD as a platform for nucleotide and protein interactions. Mutational analysis reveals that the GBD is also used to bind the GK ligand MAP1a, suggesting that this is a common mode of MAGUK complex assembly. The GK^enz^ undergoes a dramatic closing reaction upon GMP binding but the protein-bound GK^dom^ remains in the ‘open’ conformation indicating that the dramatic conformational change has been lost in the conversion from nucleotide kinase to phosphoprotein recognition domain.

## Introduction

Protein interaction domains form the backbone of cellular information processing networks [1], [2]. These small, modular sequences mediate the multitude of interactions that underlie biological regulatory pathways. Large families of protein interaction domains, such as SH3, PDZ, and PTB, have evolved, each with a particular fold and recognition code, to satisfy the demand for protein interactions [3]. Individual members of a protein interaction domain family likely evolved from a common ancestor that expanded through gene duplication events with subsequent mutations leading to functional specialization (e.g. specific binding to a particular target protein) [4], [5]. Understanding the origins of protein interaction domains could provide new insight into the function of these fundamental signaling components [6].

Here we examine the recognition mechanism of a protein interaction domain that evolved from a nucleotide kinase. The Membrane Associated Guanylate Kinase (MAGUK) family of proteins contain the Guanylate Kinase domain (GK^dom^) that diverged from Guanylate Kinase enzymes (GK^enz^) near the appearance of animals [7], [8], [9]. The GK^enz^ is part of the nucleotide kinase family of enzymes that is broadly distributed and catalyzes phosphoryl transfer from ATP to GMP [10]. The GK^dom^, in contrast, is limited primarily to metazoan MAGUK proteins; it has lost catalytic activity but gained the ability to bind proteins [8]. Thus, although GK^enz^ and GK^dom^ have high sequence and structural similarity [10], [11], [12], GK^enz^ has enzymatic activity but no known peptide ligands, whereas GK^dom^ has multiple peptide ligands but no known enzymatic activity [13], [14]. The taxonomic distributions of GK^enz^ and GK^dom^ suggest that GK^dom^ is derived from GK^enz^ leading to an evolutionary model in which GK^dom^ has lost its original function but gained a new one [7], [9].

GK^dom^-mediated protein interactions are important in a variety of cellular contexts, such as neurological synapse function, adhesion, and mitotic spindle orientation [8], [15]. In one example, the GK^dom^ from the MAGUK Discs-large (Dlg) is required for cortical recruitment and spindle orientation by Partner of Inscuteable (Pins) [15], [16]. Spindle orientation is important in many contexts, such as asymmetrically dividing *Drosophila* neuroblasts, which polarize during cell division to segregate distinct fate determinants into the daughter cells [17], [18], [19]. The Pins Linker domain (Pins^LINKER^) is sufficient for Dlg recruitment, although Pins must be phosphorylated by Aurora A [15]. Dlg, in turn, is recruited to the cell cortex through its GK^dom^ [15]. Other GK domains function in diverse physiological processes such as the formation of epithelial cell adhesions and scaffolding of the postsynaptic density at neuronal synapses [8]. These activities are mediated by numerous protein binding partners including GKAP [20], MAP1A [21], [22], GukH [23], and calmodulin [24].

Although MAGUK proteins were identified almost 20 years ago [25], [26], the structural mechanism of protein binding by the enzyme-derived GK^dom^ is unknown. Despite the importance of GK^dom^-mediated interactions, it has been unclear how protein-binding activity is accomplished from its nucleotide kinase scaffold. Are the elements that were used for nucleotide recognition used for protein recognition? To what extent has the domain been remodeled for this new function? In this work we set out to characterize the interaction between the Dlg GK^dom^ and Pins, and to determine the structural mechanism of protein recognition by the domain.

## Results

### The Dlg GK domain is a specific phosphoprotein recognition module

**Figure 1:**
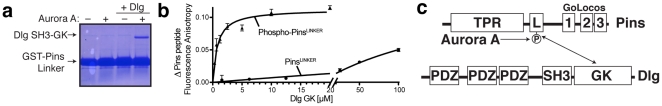
The Guanylate Kinase domain is an enzyme-derived phosphoprotein recognition domain. (A) The Dlg GK domain is a specific phosphoprotein recognition domain. A GST-pull down experiment shows that the Dlg SH3-GK region only interacts with the Pins Linker domain when it has been phosphorylated by Aurora A. (B) Change in fluorescence anisotropy of phosphorylated and unphosphorylated Pins Linker peptides as a function of Dlg GK domain concentration. The curves represent binding affinities of 0.8 µM (phosphorylated) and 206 µM (unphosphorylated). (C) Domain structure of Pins and Dlg. Pins consists of Tetratricopeptide repeats (TPR), a linker domain (L), and three GoLoco motifs (1–3). Dlg contains three PDZ domains, and SH3 domain, and the GK domain. The mitotic kinase Aurora A phosphorylates the Pins Linker domain initiating an interaction with the Dlg GK domain.

Phosphoprotein recognition modules bind specifically to the phosphorylated form of short peptide segments present in their target proteins [27]. The GK domain is the only domain from Dlg required for mitotic spindle orientation, and Dlg has been shown to interact with Pins by co-immunoprecipitation. Furthermore, the region of Pins that is required for Dlg recruitment to the cell cortex (Pins^LINKER^) must be phosphorylated by Aurora A for function [15]. We hypothesized that the Dlg GK^dom^ interacts directly with Pins^LINKER^ and that interaction might be phospho-regulated leading to the requirement for Aurora A activity. To determine if this is the case, we used purified components and compared the extent of binding to unphosphorylated and Aurora A phosphorylated Pins. Consistent with phosphorylation being a prerequisite for spindle orientation, we find that Dlg binds phosphorylated Pins, but we did not detect binding to the unphosphorylated form (**Fig. 1a**). To determine the degree to which GK^dom^ is selective for phosphorylated Pins, we monitored the steady state emission anisotropy of a rhodamine attached to a Pins^LINKER^ peptide. Using this assay, we observed that the isolated GK^dom^ has an affinity of *K*_d_ = 0.8±1 µM for phospho-Pins but >200 µM for the unphosphorylated peptide (**Fig. 1b**). Thus, the enzyme-derived Dlg GK^dom^ is highly selective for a phosphorylated peptide from its target protein (**Fig. 1c**), similar to phospho-peptide binding domains 14-3-3, WW, and FHA [27].

### Structure of the Discs large GK domain bound to Pins

To elucidate the structural mechanism of phospho-Pins recognition by the GK^dom^, we determined the structure of the complex using X-ray crystallography. The Pins^LINKER^ containing a phosphomimetic aspartic acid in place of the phosphorylated serine (S436D) was fused with the Dlg GK^dom^ and its adjacent SH3 domain, which together form a functional supermodule. Pins containing the S436D mutation fully rescues Pins function in absence of Aurora A kinase [15], indicating that it is a functional mimic for Pins binding to Dlg. To ensure that the correct binding site was used in the covalent linkage between GK and Pins, we tested if the linked complex competed against binding to Pins *in trans.* As shown in **Fig. S1a**, the affinity of Pins for the GK is significantly higher (50-fold) than for the GK-Pins fusion (this effect is dependent on the presence of the phosphomimetic residue in the Pins sequence) indicating that the *in cis* complex competes against complex assembly. Thus, we conclude that the Pins^LINKER^ in our *in cis* construct is in the same binding site as the *in trans* complex.

The structure of the GK-Pins fusion was determined using cryogenic data extending to 1.6 Å (**Table S1**) and initial phases were determined by molecular replacement using the SH3-GK structure of PSD-95 as a search model. The crystals contained a single Dlg-Pins in the asymmetric unit. The final model has excellent geometry and comparison with the crystallographic data yields an R/R_free_ of 0.23/0.25. Several stereo views of the Pins Linker electron density calculated from a composite omit map are shown in **Fig. S1b,c**.

### The vestigial GMP binding domain has been co-opted for protein binding

**Figure 2:**
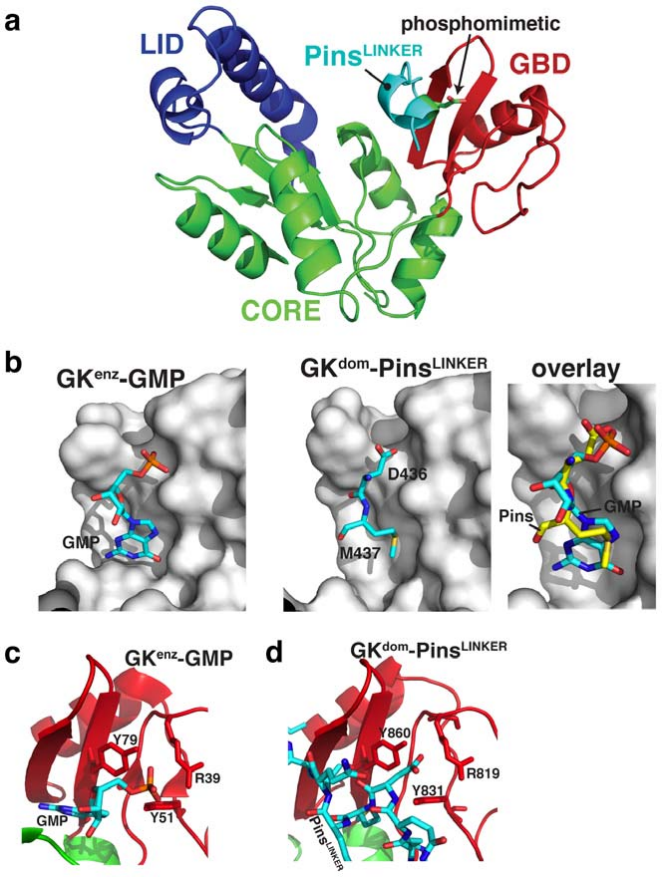
Phospho-Pins binds the vestigial GK GMP binding domain. (A) Structure of Dlg-Pins. LID, CORE, and GBD subdomains, as described for the GK enzyme, are highlighted. The phosphomimetic D436 is shown in the Pins Linker. (B) The GMP nucleotide-binding pocket. The left panel shows the interaction of GMP with the GBD subdomain taken from the yeast Guanylate Kinase structure 1EX7 [10]. The right panel shows only those residues of the Pins Linker domain that occupy the nucleotide binding pocket to show how the phosphorylated residue (D436 in the structure) and M437 mimic GMP interactions. An overlay of GMP and Pins residues 436 and 437 on the GK domain is also shown. (C) GK enzyme phosphorecognition. Three residues form the primary contacts with the GMP phosphate, as shown from the yeast Guanylate Kinase structure 1EX7 [10]. (D) GK domain phosphorecognition. The residues that contact the phosphate in the GK enzyme are conserved in the domain and assume an identical configuration.

How has the nucleotide kinase scaffold been adapted to bind phosphoproteins? The GK^enz^ is divided into three subdomains, the LID, CORE and GMP-Binding Domain (GBD) [10], [28], and each has a structural analogue in the GK^dom^ (**Fig. 2a**) [11], [12]. The LID domain contains many of the ATP-binding residues in GK^enz^ and is the region of the protein that has diverged most significantly in GK^dom^ (GK^dom^ does not bind ATP or GMP). The GBD contains most of the GMP binding pocket, and the CORE connects the LID to the GBD.

Pins interacts nearly entirely through contacts with the vestigial GBD in GK^dom^ (**Fig. 2a**). In GK^enz^, the guanosine base portion of GMP sits in a deep pocket on the GBD whereas the phosphate is exposed on the GBD surface. The GK^enz^ residues that contact GMP are well conserved in GK^dom^, and this binding site is used by Pins. Two Pins residues occupy the GMP binding pocket: a methionine (M437) whose side chain inserts into the pocket that binds the GMP base, and the phosphoserine (D436 in the structure) that sits in place of the nucleotide's sugar and phosphate (**Fig. 2b**). In GK^enz^, the guanosine phosphate is recognized by three side chains, two tyrosines and an arginine oriented towards the phosphate oxygens (**Fig. 2c**). These three residues are conserved in GK^dom^ and assume an identical configuration in contacting the phosphorylated Pins residue (**Fig. 2d**).

**Figure 3:**
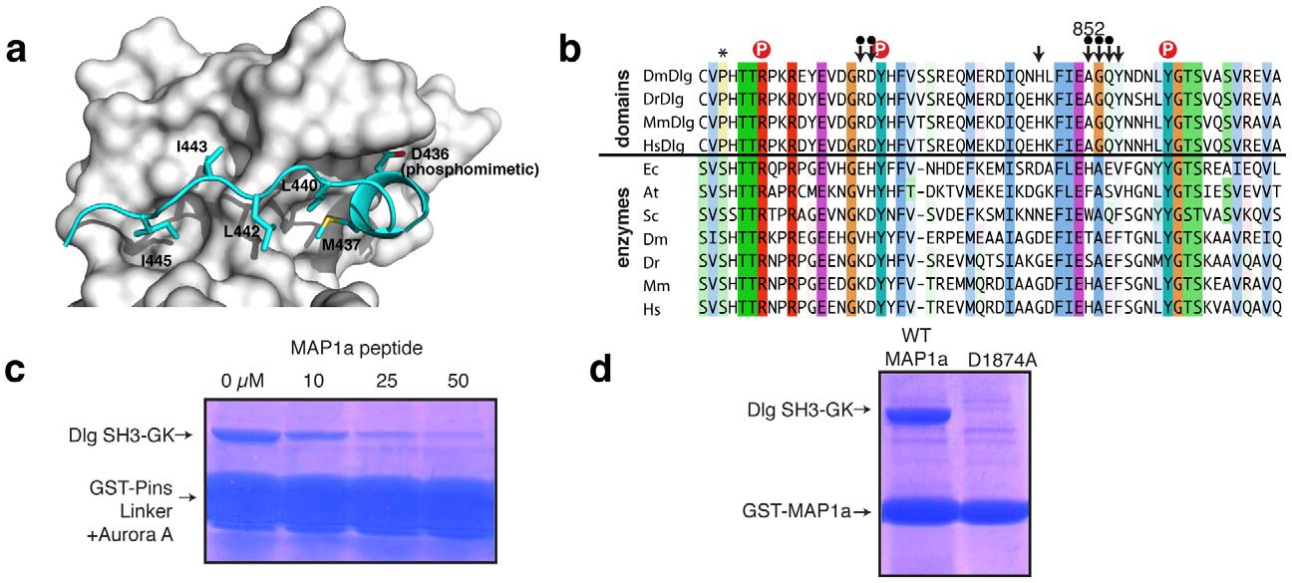
Extensive interactions between the Pins Linker and the GK domain GBD. (A) Pins Linker makes extensive GBD contacts outside of the nucleotide binding site. The two residues that occupy the nucleotide binding site, D436 and M437, are shown along with the hydrophobic residues that form interactions along the GBD. (B) Alignment of GK enzyme and domain GMP-binding domains. (*Ec = Escherichia coli; Sc = Saccharomyces cerevisiae; At = Arabidopsis thaliana; Dm = Drosophila melanogaster; Mm = Mus musculus; Hs = Homo sapiens*). Residues that contact Pins are indicated by an arrow (a dot above the arrow indicates backbone contacts). The conserved alanine residue (number 852 in Discs large) is highlighted. Residues that contact the phosphate are shown by a red circle. Asterisk indicates residue that induces functional switch from enzyme to domain [15]. (C) MAP1a residues 1862–1883 compete with Pins for binding to the GK domain. A GST-fusion of the Pins linker region that was incubated with Aurora A efficiently pulls down the Dlg SH3-GK module, but this interaction is displaced by a MAP1a peptide. (D) Aspartic acid 1874 within MAP1a residues 1862–1883 is required for interaction with the GK domain as assessed by GST-pull down.

Pins also makes extensive contact with the GK^dom^ GBD outside of the nucleotide binding pocket. Overall, 11 Pins residues contact the GK^dom^ GBD yet only two interact with the GMP binding pocket. The remaining residues assume an extended conformation that sits across the interior face of the GBD (**Fig. 3a**). Four Pins residues (two leucines and two isoleucines) form hydrophobic contacts with the GBD. Surprisingly, most of the atoms that Pins contacts on the GK^dom^ GBD are conserved with GK^enz^ (**Fig. 3b**), indicating that the GBD was not extensively remodeled to support a protein binding function. The lack of significant changes in the GBD is consistent with the recent finding that only a single mutation is sufficient to convert extant GK^enz^ into a functional GK^dom^ [29]. A notable exception is a variable residue in GK enzymes that is highly conserved in GK domains (alanine 852). One of the isoleucines from Pins sits in the pocket created by this residue and would clash with the longer sidechains found in the enzymes. The high degree of conservation at this position in GK domains from Dlg family members (**Fig. 3b**) suggests that the GBD might be a common interaction surface, consistent with previous NMR evidence that the protein MAP1a interacts with the GK^dom^ GBD from PSD-95 [22].

To test if MAP1a and Pins do indeed utilize the same binding mode on the GK^dom^, we performed a competition experiment evaluating whether a MAP1a peptide could compete with Pins binding to GK^dom^. The GK^dom^ bound to an Aurora A phosphorylated Pins is efficiently competed away by the MAP1a peptide (**Fig. 3c**). The MAP1a sequence does not have an obvious phosphorylatable residue and does not require phosphorylation to bind GK^dom^. We hypothesized that an acidic residue present in the MAP1a sequence may function as a natural phosphomimetic. Mutation of D1874 in MAP1a completely abrogates binding (**Fig. 3d**), consistent with this hypothesis. Thus, we conclude that MAP1a and Pins compete for binding to GK^dom^ and MAP1a my bypass the requirement for phosphorylation using a phosphomimetic residue.

### GK domain structural interactions are required for protein binding and spindle orientation

**Figure 4:**
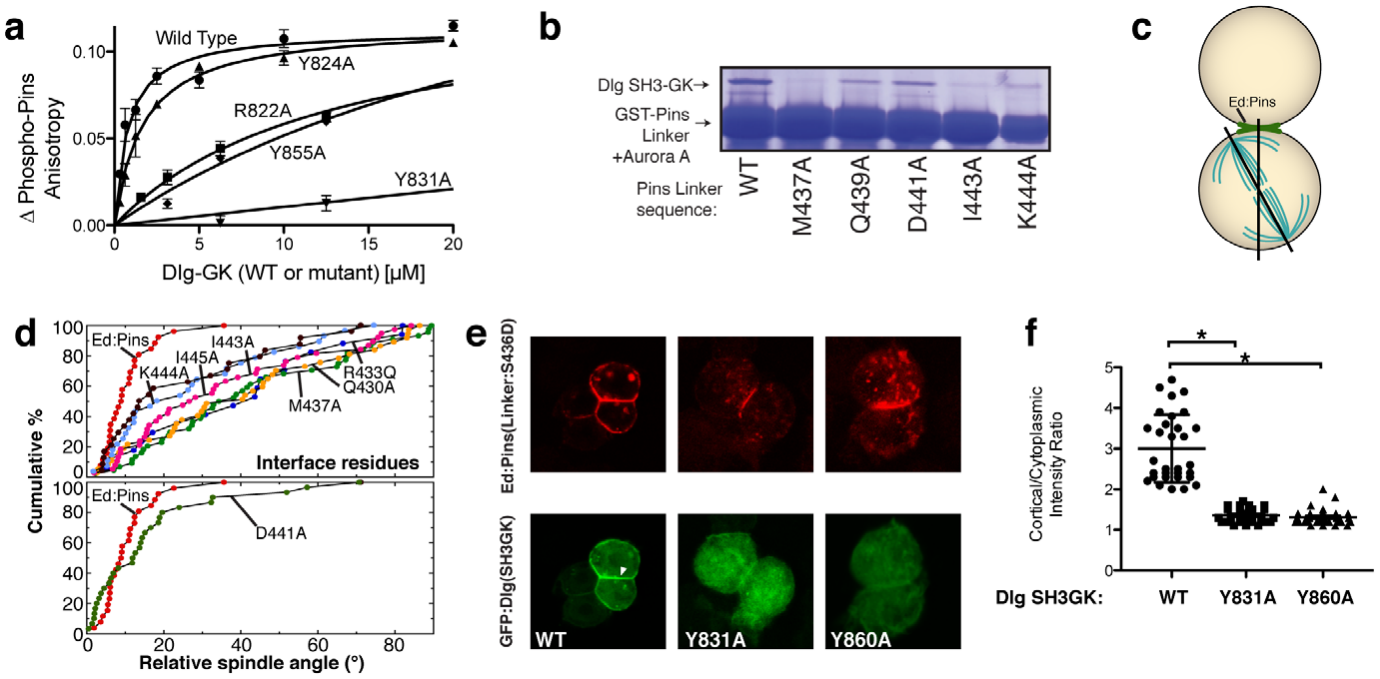
Dlg GK domain contacts with Pins Linker are required for mitotic spindle orientation. (A) GK domain mutations in residues at the binding interface lower the affinity for a phosphorylated Pins Linker peptide. The change in anisotropy of a rhodamine attached to the peptide is shown as a function of wild-type or the indicated mutant Dlg GK domains. (B) Pins Linker domain mutations in residues at the binding interface lower the affinity for the GK domain. GST-fusions of Pins linker regions containing the indicated mutations were incubated with Aurora A and their ability to pull-down the Dlg SH3-GK were assessed. (C) Schematic of *Drosophila* S2 cell induced polarity spindle orientation assay. Clustered cells polarize Echinoid-Pins (Ed-Pins) to sites of cell-cell contact. Ed-Pins with mutant Linker domains were assessed for their ability to orient the spindle by measuring the angle between the center of the crescent and the mitotic spindle. (D) Cumulative percentage plots of spindle orientation by Ed-Pins mutants. These plots show the cumulative percentage of cells that have a spindle angle below a particular value (x-axis). Cells expressing wild-type Ed-Pins have predominantly small angles between the Ed-Pins crescent and the spindle whereas cells expressing defective Ed-Pins have random distributions (diagonal distribution in the cumulative percentage plot). Mutation of Pins Linker residues that contact the GK domain (top panel) leads to loss of spindle orienting activity. In contrast, mutation of a residue that faces away from the domain (D441A) has little effect. (E) GK^dom^-Pins^LINKER^ interactions are required for GK^dom^ recruitment to induced Ed-Pins crescents in *Drosophila* S2 cells. A GFP-fusion of the Dlg SH3-GK domain localizes to Ed-Pins crescents (white arrowhead). Mutation of Y831 or Y860 in the GK domain GBD to alanine prevents recruitment. (F) Quantification of GK^dom^ recruitment to induced Ed-Pins crescents in *Drosophila* S2 cells for the data in panel E. The ratio of the cortex and the cytoplasm for the GFP-Dlg SH3-GK signal is shown. Error bar represents one standard deviation. Asterisks represent p<0.001 using ANOVA with Dunn's post-hoc test.

To determine if the interactions identified in the GK^dom^-Pins^LINKER^ complex are functionally important, we examined their role both in protein binding in vitro and functionally using a spindle orientation assay we recently developed [15]. For the protein binding assay, we used fluorescence polarization, as in **Fig. 1c**. We examined whether mutations in the GK^dom^ predicted to be important for binding Pins would result in a lower affinity. As shown in **Fig. 4a**, we observed that GK contact residues lowered the affinity for Pins ranging from 13 µM to 700 µM compared to the affinity for the wild-type sequence of 0.8 µM (**Fig. 1c**). Mutation of a neighboring tyrosine residue (Y824) that does not make contact with Pins did not significantly alter affinity (**Fig. 4a**). We also tested the role of Pins residues that contact Dlg in stabilizing their interaction (**Fig. 4b**). We observed that the buried methionine that occupies the GMP binding site (M437) is critical for the interaction, as are another buried hydrophobic residue (I443A), and a residue that forms a salt bridge (K444A). Other residues that do not contact GK^dom^ (Q439 and D441) have little effect.

We also examined whether mutations predicted to disrupt the GK^dom^-Pins^LINKER^ complex affected spindle orientation using a cultured cell assay. In this assay, Pins crescents are induced in cultured *Drosophila* S2 cells by fusing Pins to the adhesion protein Echinoid (Ed). Clusters of adhered cells restrict Ed and the attached Pins protein to the area of cell-cell contact, and during mitosis the spindle becomes aligned with the center of the Ed-Pins crescent (**Fig. 4c**). As the interaction of Dlg with Pins is required for spindle orientation, the induced polarity assay can be used to assess the relative importance of GK^dom^-Pins^LINKER^ interactions to biological function.

We made mutations in a number of Pins^LINKER^ residues and tested the ability of the resulting Ed-Pins fusions to orient the spindle. Mutation of critical contact residues in the binding interface results in reduced spindle orienting activity (**Fig. 4d**). In contrast, mutation of an aspartic acid that points away from the domain had little effect on spindle positioning. Likewise, mutation of GK residues that interact with Pins^LINKER^ in the structural model prevented GFP-tagged Dlg GK^dom^ from being recruited to the Pins crescent (**Fig. 4e,f**). Together, these results demonstrate that the interactions present in the GK^dom^-Pins^LINKER^ structural model are required for Pins-Dlg interaction and proper spindle orientation.

### The GK enzyme closes upon ligand binding but the domain does not

**Figure 5:**
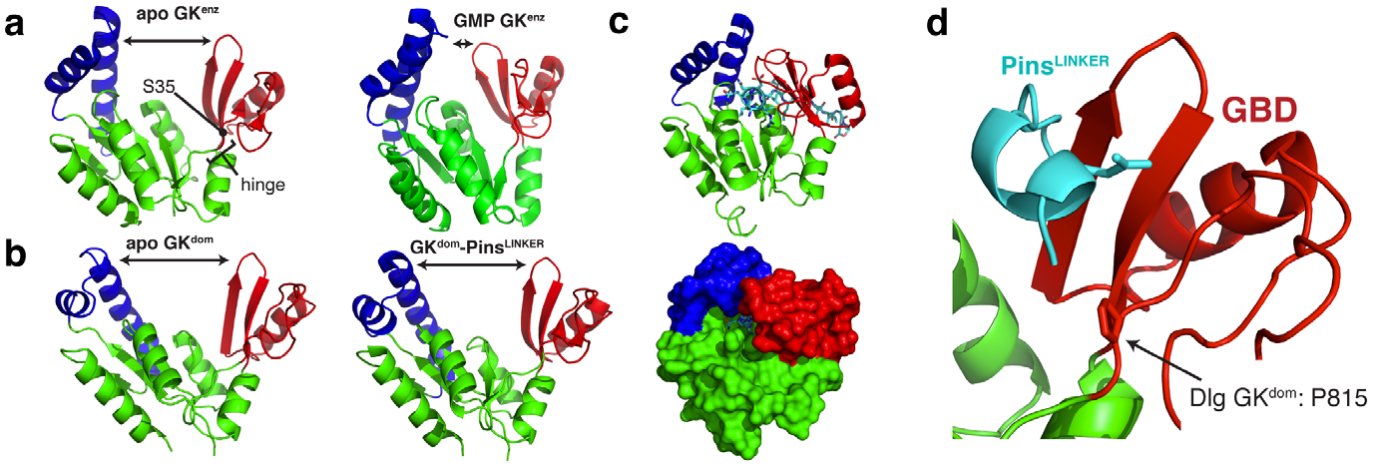
Loss of a ligand-induced conformational change in the GK domain. (A) Nucleotide binding to the GK enzyme induces a large conformational change. GMP binding causes a transition from an “open" conformation to a “closed" one allowing for long range communication between the ATP and GMP binding sites. The “hinge" region that undergoes dihedral angle changes during closing is shown. Recently it was found that mutation of a serine residue (S35) in this region to proline is sufficient to convert enzyme to domain [29]. Structures 1EX6 and 1EX7 are shown [10]. (B) The GK domain conformation does not change upon ligand binding. Unlike the GK enzyme, the GK domain does not assume a “closed" conformation upon Pins binding to the GBD. (C) GK closing is incompatible with Pins binding to the GBD. Ribbon and surface representations are shown of Pins aligned to the closed GK enzyme structure (1EX7) showing that closing would cause dramatic steric overlaps with the protein ligand. (D) A critical serine to proline mutation is in the GK “hinge" region. Mutation of a conserved GK enzyme serine is sufficient to convert it into a GK domain [29]. The position of this proline, which lies in the region that undergoes dramatic dihedral angle changes in GK enzyme, is shown in a ribbon representation.

In GK^enz^ nucleotide binding causes a large conformational change [10], [28] and we sought to determine if ligand binding to GK^dom^ causes a similar effect. In the absence of nucleotide GK^enz^ exists in an “open" structure where both the ATP and GMP binding sites are exposed but nucleotide binding causes a transition to a “closed" form that brings the two terminal phosphates close to one another (**Fig. 5a**) [10], [28]. The long range communication between ATP and GMP binding sites is mediated primarily by GMP binding as the GMP-bound structure is nearly completely in the closed conformation. The structures of all apo GK^dom^s determined to date share the open conformation of the unbound enzyme [11], [12], [30], [31]. However, in contrast to the enzyme, GK^dom^ remains open upon ligand binding as the Dlg GK^dom^ in complex with Pins remains in the open form (**Fig. 5b**). To investigate the source of the difference in binding associated dynamics between enzyme and domain, we aligned Pins onto the closed enzyme structure to examine the effect closing would have on protein binding (**Fig. 5c**). In GK^enz^, the closed conformation creates a binding pocket that nearly completely engulfs GMP, creating additional interactions with the CORE domain. As Pins is significantly larger than GMP, the closed form would create a large number of steric overlaps that would preclude binding. Thus, the ligand-induced conformational change is necessary for GK^enz^ catalysis but would be detrimental to GK^dom^ protein binding.

## Discussion

The proliferation of protein interaction domains in genomes emphasizes the critical role these components play in cellular signaling pathways [32]. Gene duplication events have driven the dramatic growth of protein interaction domain families suggesting that these families share a common ancestor [5], yet the nature of these ancestral proteins is rarely known. MAGUK GK domains are an exception as the prevalence of GK enzymes and limited distribution of GK domains suggests that the common ancestor to MAGUKs was a nucleotide kinase [7], [9]. While the mechanism of the transition from the ancestral kinase to the MAGUK domain remains to be characterized, a single mutation from serine to proline converts extant GK^enz^ into a functional GK^dom^, and this mutation also alters GK dynamics [29]. Nevertheless, a critical piece of information has been missing: the mechanism of protein binding by GK^dom^. In this work we have addressed this gap in understanding by determining the structure of a Dlg-Pins fusion protein. A recent structure of the mammalian orthologues of the proteins studied here (Dlg and LGN) has recently been solved [33], corroborating our observations.

### Use of an existing nucleotide binding surface for protein recognition

The structure of Dlg-Pins reveals that the binding surface for Pins on the GK^dom^ was not extensively remodeled to support a protein binding function. The core of the Pins interaction utilizes the nucleotide binding pocket, which is nearly identical to that found in extant enzymes. Pins binding is regulated by phosphorylation, and the nucleotide phosphate binding site could be used “as-is" for this purpose. Not all GK^dom^ ligands are known to be phosphorylated (e.g. MAP1a) and we expect that their interactions with the phosphate binding site are critical such that acidic residues may take the place of the phosphorylated serine in these binding partners.

A key difference between protein and nucleotide binding is that Pins makes extensive contact with the GBD outside of the nucleotide binding pocket. For the most part, however, these interactions are with backbone atoms or with residues that are conserved between enzyme and domain. Thus, protein binding makes use of a surface that likely existed 600 million years ago in the common ancestor of GK^enz^ and GK^dom^ suggesting that Pins adapted to bind this surface as opposed to the GBD adapting to Pins. This model would require a minimum number of GK mutations for neofunctionalization, consistent with the recent finding that a single serine to proline mutation within the GK hinge region (**Fig. 5d**) disrupts catalytic activity but leads to a gain of protein binding.

### Loss of protein conformational dynamics correlated with neofunctionalization

Nucleotide binding causes a dramatic GK^enz^ conformational change in which ATP and GMP binding sites that were distant in the apo form are brought together [10], [28]. Interestingly, the serine to proline mutation that switches GK function from enzyme to domain also disrupts the nucleotide induced conformational change [29]. The structure presented here explains why this may be important–closing of the GK, while critical for enzyme function as it brings the two phosphates together, would cause significant steric overlap with the protein ligand. Thus, the key mutation that converts a nucleotide kinase to a phosphoprotein recognition domain apparently did so not by creating a new protein binding surface, but by altering protein dynamics. In general, protein dynamics could be a common property exploited by evolution [34], [35] as minimal mutations could alter protein movements compared to the extensive sequence changes that can be required to build new binding surfaces.

## Methods

### Plasmid construction and protein purification

An *in-cis* Dlg/Pins fusion was constructed by simultaneous ligation of independent PCR products (Dlg = residues 598–975 with BamHI/XbaI restriction sites and Pins = residues 411–460 with XbaI/XhoI restriction sites) into BamHI/XhoI digested pBH4 plasmid backbone [36]. Using standard PCR-based site directed mutagenesis, Pins was subsequently mutated (S436D) to create a phosphomimetic sequence necessary for Dlg binding. In addition, residues 679–766 of Dlg (known as the ‘Hook’ domain) were removed by deletional mutagenesis PCR to facilitate crystallization.

Protein expression and purification from the pBH4 plasmid were as previously described [37], [38]. Briefly, the resulting pBH4-ΔHook/Pins^S436D^ plasmid was transformed into BL21(DE3) competent *E. coli* and grown as an overnight starter culture under ampicillin (100 µg/ml) selection. Six liters of LB were inoculated with starter culture, allowed to grow to and OD_600_ = 0.7, and induced with the addition of 200 µM IPTG overnight at 20°C. Protein purification was carried out using sequential NiNTA affinity, anion exchange, and size exclusion chromatographies. The ΔHook/Pins^S436D^ eluted as a predicted monomer from the size exclusion column, and its purity was assessed to be >95% by coomassie staining of an SDS-PAGE gel. Protein was concentrated to 32 mg/ml using Vivaspin concentrators (Sigma Aldrich, St. Louis, MO), flash frozen in liquid nitrogen, and stored at −80°C in buffer (20 mM Tris, pH 7.5, 50 mM NaCl, 5% glycerol, 2 mM DTT).

### Crystallization, structure determination, and refinement

Crystals of the ΔHook/Pins^S436D^ fusion protein were grown by vapor diffusion from a 1∶1 (v/v) mixture of protein (32 mg⋅ml^−1^) and well solution (2 M NaCl plus 0.1 M NaOAc, pH 4.6). Crystals appeared within 2–3 days and reached maximum dimensions of 0.5×0.2×0.2 mm. Crystals formed in the C222_1_ orthorombic space group with a single ΔHook/Pins^S436D^ molecule in the asymmetric unit. Prior to data collection, crystals were cryoprotected for 1 minute in well solution supplemented with 25% glycerol and then flash frozen in liquid nitrogen for data collection at 100 K. A native data set was collected using remote access data collection and was performed on the 5.0.1 beamline at Advanced Light Source (Berkeley, CA). Diffraction data were scaled and indexed spacing using HKL2000 [39]. The structure of ΔHook/Pins^S436D^ was determined using molecular replacement with the Phaser software in CCP4 [40] using the rat PSD-95 structure (PDB ID 1KJW) as a search model [11]. Model building was completed using Coot [41] and refined using Refmac [40]. TLS was used to model thermal displacements with four groups (Dlg residues 602–779, 780–971, and Pins residues 434–442, 443–447). The final model excludes Dlg residues 614–622 and 650–657 within the SH3 domain loops due to insufficient electron density. Additionally, Pins^LINKER^ residues 435–441, including the well-resolved S436D residue, were sufficiently modeled in the final structure. All structural images were made using PyMol (Delano Scientific, San Carlos, CA).

### Cell culture and Echinoid cell-adhesion assays

Maintenance of S2 cells, construction of expression plasmids (including Echinoid fusion sequences), and cell adhesion assays were as previously described [15], [42]. Briefly, S2 cells were transfected using the Effectene reagent (Qiagen, Germantown, MD) with 0.4–1 µg total DNA for 24–48 hours. Protein expression was then induced by the addition of 500 µM CuSO_4_ for 24 hours. Cell adhesion clustering was induced by constant rotation at ∼175 RPM for 1–3 hours.

Detailed immunostaining procedures have also been described [15], [43]. Briefly, cells were fixed in 4% paraformaldehyde for 20 minutes, washed, and incubated with primary antibodies overnight at 4°C. Slides were subsequently washed and fluorescently-linked secondary antibodies were added for 2 hours at room temperature. Finally, slides were again washed and mounted using Vectashield Hardset medium (Vector Laboratories, Burlingame, CA). All images were collected using a Leica SP2 confocal microscope with a 60×1.4 NA lens.

### GST Pulldowns

Pulldowns were as previously described [44], [45]. Briefly, GST-tagged Pins (residues 399–466) or MAP1a (residues 1862–1883) were absorbed to glutathione agarose for 30 minutes at 4°C and subsequently washed 3 times with PBS. To produce S436-phosphorylated Pins bait, GST-Pins was then incubated in the presence or absence of 0.5 µg Aurora-A (Millipore) in kinase buffer (20 mM Tris, pH 8, 50 mM NaCl, 5 mM MgCl2, 1 mM EDTA, 1 mM DTT, and 100 µM ATP) for 30 minutes at room temperature. Subsequently, 50 µg of Dlg was added for 1 hour at 4°C. Reactions were washed (20 mM Tris, pH 7.5, 100 mM NaCl, 5 mM MgCl2, and 0.5% NP-40), and samples were analyzed using coomassie blue staining.

### Fluorescence anisotropy

Fluorescence anisotropy experiments were conducted using an ISS PC1 Photon Counting Spectrofluorometer (Fluorescence & Analytical Instrumentation, Champaign, IL, USA) with polarization filters. Pins peptides (residues: 427–445) were synthesized with and without a pSer at the residue corresponding to S436 and labeled with an N-terminal tetramethylrhodamine (TMR) dye and purified to >95% purity via HPLC. TMR-labeled peptides were diluted in assay buffer (20 mM Tris, pH 8, 100 mM NaCl, and 1 mM DTT) to 0.5 µM and 10 iterative anisotropy measurements were conducted and averaged. Increasing concentrations of Dlg constructs were mixed with 0.5 µM peptide and measured individually under identical measurement conditions. Data were collected using the Vinci software package (v1.5). Saturation binding curves and affinity calculations were conducted using nonlinear regression analysis in the Prism software suite (GraphPad Software, La Jolla, CA, USA).

## Supporting Information

Figure S1 (A) The “in cis" Pins Linker competes with the in trans interaction. The change in anisotropy of a phosphorylated rhodamine labeled Pins Linker peptide is shown as a function of three different Dlg SH3GK domains. One domain contains the Pins Linker fused to the SH3GK with a phosphomimetic residue (the crystallography construct) and has significantly lower affinity (14 µM) than the other proteins (0.3 µM; SH3GK alone or SH3GK fused to Pins without the phosphomimetic). (B,C) Pins Linker electron density. Stereo views of the electron density from a composite omit map contoured at 1.3 sigma is shown in two different orientations in (B) and (C).
(PDF)

Table S1 X-ray refinement statistics.
(DOC)
