# Supplementary figures and images for: Structure of an Enzyme-Derived Phosphoprotein Recognition Domain
Source: PLoS One. 2012 Apr 24;7(4):e36014. doi: 10.1371/journal.pone.0036014 (PMC3335814; doi:10.1371/journal.pone.0036014)

Figure S1

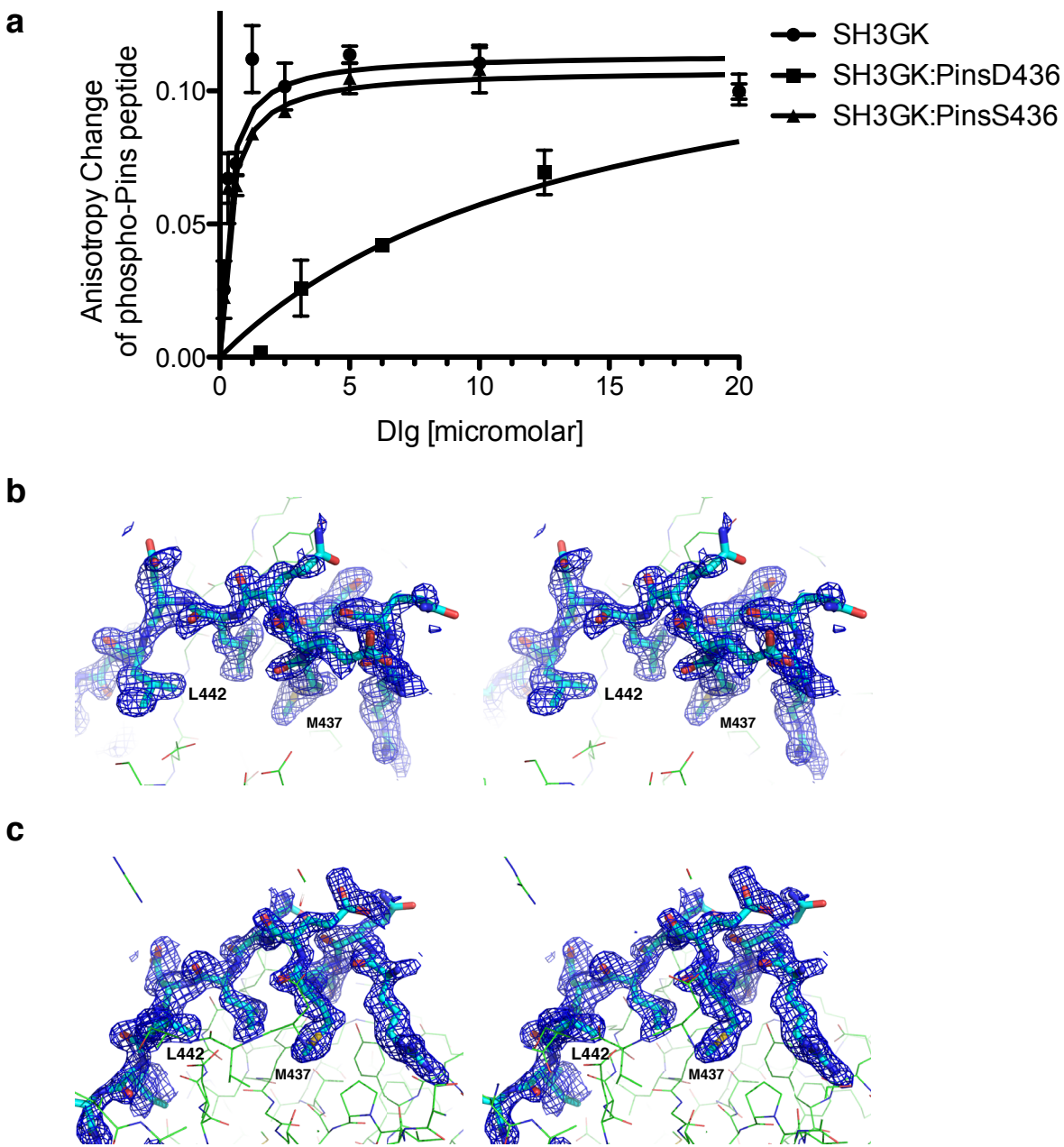

Supplement: Figure S1 — (A) The “in cis" Pins Linker competes with the in trans interaction. The change in anisotropy of a phosphorylated rhodamine labeled Pins Linker peptide is shown as a function of three different Dlg SH3GK domains. One domain contains the Pins Linker fused to the SH3GK with a phosphomimetic residue (the crystallography construct) and has significantly lower affinity (14 µM) than the other proteins (0.3 µM; SH3GK alone or SH3GK fused to Pins without the phosphomimetic). (B,C) Pins Linker electron density. Stereo views of the electron density from a composite omit map contoured at 1.3 sigma is shown in two different orientations in (B) and (C). (PDF) [file pone.0036014.s001.pdf]
