# Supplementary material for: Structure of an Enzyme-Derived Phosphoprotein Recognition Domain
Source: PLoS One. 2012 Apr 24;7(4):e36014. doi: 10.1371/journal.pone.0036014 (PMC3335814; doi:10.1371/journal.pone.0036014)
Supplement: Table S1 — X-ray refinement statistics. (DOC) [file pone.0036014.s002.doc]

| Table S1 X-ray refinement statistics | |
| --- | --- |
| Resolution (Å) | 30-1.6 |
| Linear R (mean, 1.6-1.66 Å shell) | 4% / 37% |
| Reflections (total, 1.6-1.66 Å shell) | 47,576 / 4547 |
| Completeness (total, 1.6-1.66 Å shell) | 99.2% / 95.9% |
| *R*_work_/*R*_free_ | 23/25 |
| Number of atoms |  |
| Protein *(Dlg)* | 2095 |
| Ligand *(Pins)* | 118 |
| Water | 240 |
| B-factors |  |
| Protein *(Dlg)* | 35.2 |
| Ligand *(Pins)* | 48.0 |
| Water | 39.1 |
| R.m.s deviations |  |
| Bond lengths (Å) | 0.017 |
| Bond angles (°) | 1.7 |
